# Efficient phenotypic sex classification of zebrafish using machine learning methods

**DOI:** 10.1002/ece3.5788

**Published:** 2019-11-11

**Authors:** Shahrbanou Hosseini, Henner Simianer, Jens Tetens, Bertram Brenig, Sebastian Herzog, Ahmad Reza Sharifi

**Affiliations:** ^1^ Department of Animal Sciences University of Goettingen Goettingen Germany; ^2^ Center for Integrated Breeding Research University of Goettingen Goettingen Germany; ^3^ Institute of Veterinary Medicine University of Goettingen Goettingen Germany; ^4^ Max Planck Institute for Dynamics and Self‐Organization Goettingen Germany; ^5^ Department for Computational Neuroscience 3rd Physics Institute‐Biophysics University of Goettingen Goettingen Germany

**Keywords:** color, machine learning, sex classification, temperature, zebrafish

## Abstract

Sex determination in zebrafish by manual approaches according to current guidelines relies on human observation. These guidelines for sex recognition have proven to be subjective and highly labor‐intensive. To address this problem, we present a methodology to automatically classify the phenotypic sex using two machine learning methods: Deep Convolutional Neural Networks (DCNNs) based on the whole fish appearance and Support Vector Machine (SVM) based on caudal fin coloration. Machine learning techniques in sex classification provide potential efficiency with the advantage of automatization and robustness in the prediction process. Furthermore, since developmental plasticity can be influenced by environmental conditions, we have investigated the impact of elevated water temperature during embryogenesis on sex and sex‐related differences in color intensity of adult zebrafish. The estimated color intensity based on SVM was then applied to detect the association between coloration and body weight and length. Phenotypic sex classifications using machine learning methods resulted in a high degree of association with the real sex in nontreated animals. In temperature‐induced animals, DCNNs reached a performance of 100%, whereas 20% of males were misclassified using SVM due to a lower color intensity. Furthermore, a positive association between color intensity and body weight and length was observed in males. Our study demonstrates that high ambient temperature leads to a lower color intensity in male animals and a positive association of male caudal fin coloration with body weight and length, which appears to play a significant role in sexual attraction. The software developed for sex classification in this study is readily applicable to other species with sex‐linked visible phenotypic differences.

## Graphical abstract

This study demonstrates the high ambient temperatures leads to masculinization and lower color pattern intensity in male animals, and a positive association of male caudal fin coloration with zootechnical parameters, which appears to play a significant role in sexual selection.

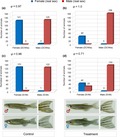

## 1 INTRODUCTION

Sexual dimorphism between males and females such as sexually dichromatism can play an important role in sexual attraction for a potential mate (Singh & Nüsslein‐Volhard, 2015). Sexual attraction with respect to color has been observed in some fish species in previous studies (Gronell, 1989; Kraak, Bakker, & Mundwiler, 1999): Mating preference in female *Chrysiptera cyanea* is correlated with intensity of orange caudal fin coloration in males during courtship periods, and reproductive success in *Gasterosteus aculeatus* increased with redness of male throat.

Coloration can therefore be used in sexually dichromatic species for sex determination. Zebrafish have emerged as a well‐established vertebrate model organism for studies of biology, genetics, embryonal development, diseases, drug screening, and environmental effect (Nowik et al., 2015; Ribas et al., 2017). This animal possesses sexual plasticity, meaning that it changes its sex depending on environmental factors (Kobayashi, Nagahama, & Nakamura, 2013), which makes it a suitable organism for studying the environmental effects on phenotypic plasticity in fish populations. Temperature is one of the most important environmental factors affecting the animal's phenotype. Previous studies reported that high temperature leads to an increase in the proportion of males in zebrafish (Abozaid, Wessels, & Hörstgen‐Schwark, 2011; Hosseini, Brenig, Tetens, & Sharifi, 2019; Hosseini, Ha, et al., 2019; Ribas et al., 2017). The sex ratio can influence population dynamics leading to a reduction in genetic variation and loss of heterozygosity, which increases inbreeding and adversely affects fitness traits resulting in the risk of extinction, particularly in small and isolated populations (Brown et al., 2015). Furthermore, a recent study demonstrated that the effect of high temperature in heat‐exposed zebrafish leads to a loss of pigmentation intensity (Ribas et al., 2017). Generally, the intensity of color in fish is regulated by genetic factors and controlled through neurohormonal mechanisms (Hutter, Hettyey, Penn, & Zala, 2012; Price, Weadick, Shim, & Rodd, 2008). Beyond the genetic regulation of color intensity, variation in coloration can be influenced by environmental factors such as temperature and light (Price et al., 2008).

In zebrafish, males show a slightly more intense yellow coloration compared to females, which is thought to be important for sexual attraction (Hutter, Penn, Magee, & Zala, 2010; Nüsslein‐Volhard & Singh, 2017; Singh & Nüsslein‐Volhard, 2015). Furthermore, previous studies showed that female zebrafish are able to visually discriminate sexes, that is, they recognize males based on their yellow coloration, particularly during courtship and spawning (Hutter et al., 2012; Hutter, Zala, & Penn, 2011). However, for human observers the differences in coloration between male and female zebrafish are often difficult to discriminate due to minor sexual dimorphism in body color in this species (Hutter et al., 2012). The conventional guidelines for sex determination in zebrafish by human perception include sex‐related differences in various features such as color, shape, behavior, and genital papilla (McMillan, Géraudie, & Akimenko, 2015). For example, females often have a round shape and protruding abdomen compared to males, but not all females possess an obviously distended abdomen (Hutter et al., 2010; McMillan et al., 2015). Furthermore, sex determination using coloration in zebrafish is difficult because there is interindividual variation in color, and in some cases, males and females exhibit similar body coloration (McMillan et al., 2015). However, a precise sex determination of adult fish can be performed using microscopic examination by dissecting gonad tissue, which requires killing animals (Abozaid et al., 2011), is labor‐intensive and requires biological expertise. Hence, the current approaches for sex determination in zebrafish are subjective and not sufficiently reliable, and highly time‐consuming (McMillan et al., 2015).

In recent years, machine learning has emerged as a promising technique for data processing in life science and computational biology (Angermueller, Pärnamaa, Parts, & Stegle, 2016; Liakos, Busato, Moshou, Pearson, & Bochtis, 2018), particularly in clustering biological images and phenotypic classification (Grys et al., 2016; Jeanray et al., 2015). Deep learning models, a relatively new branch of machine learning, consist of multiple processing layers to learn representations of large datasets and have dramatically advanced and improved the state‐of‐the‐art in various research fields (LeCun, Bengio, & Hinton, 2015; Liakos et al., 2018; Min, Lee, & Yoon, 2017). Convolutional neural networks (CNN), a deep learning architecture, recently surpassed human‐level accuracy especially for image recognition and classification (Grys et al., 2016).

The main goal of this study was to develop an efficient technique for sex determination of zebrafish using two fully automatic machine learning methods: Deep Convolutional Neural Networks (DCNNs) based on body color and pattern, and Support Vector Machine (SVM) based on caudal fin color only. We first applied DCNNs to determine the sex from the image of the whole fish body. We secondly applied SVM using the color distribution of the caudal fin pictures to automatically determine the sex of zebrafish. Both approaches used RGB images as an input and were trained in a supervised manner to classify the sex of an individual using image analysis. The estimated color intensity based on SVM was then used to quantify the degree of association between coloration and body weight and length. To further demonstrate the utility of the approaches, the SVM technique was used to quantify the impact of high water temperature on color intensity of the caudal fins of different sexes. This technique can be used in various science areas, in which sexual plasticity and its ecological relevance will be the focus of scientific research.

## 2 MATERIALS AND METHODS

### 2.1 Ethics statement

All procedures in this study were in strict accordance with the German animal welfare act and national and international recommendations. This study was approved by the University of Goettingen committee for the care and use of animals (File number E3‐17). The broodstocks were kept in the recirculation systems of aquaculture facilities according to the approved institutional guidelines on the use of animals for research purposes (Abozaid et al., 2011).

### 2.2 Experimental design and phenotypic measurements

We designed a treatment–control study to examine the effect of high water temperature during the critical period of zebrafish embryogenesis on sex ratio, color intensity, body weight and length, and their associations. For this, equal proportions of fertilized eggs from full‐sib families of Singapore strain (Hosseini, Ha, et al., 2019; Von Hertell, Hörstgen‐Schwark, Langholz, & Jung, 1990) were exposed to low (28°C) and high temperatures (35°C) during 5–24 hr post‐fertilization (hpf; Hosseini, Ha, et al., 2019). The standard rearing temperatures for zebrafish are between 26–29°C with an optimum of 28°C (Detrich, Zon, & Westerfield, 2004; Ribas & Piferrer, 2014). The high temperature applied in the temperature‐exposed group of this study is in line with previously reported studies (Hosseini, Brenig, et al., 2019; Hosseini, Ha, et al., 2019; Abozaid et al., 2011). The temperature of heat‐exposed groups was changed gradually in this experiment. Two weeks after hatching, the temperature treatment and control groups of different families were separately mixed in bigger tanks to eliminate the effect of population density within tanks and kept until sexual maturity (90 days post‐fertilization). After maturation, total length and body weight of all individuals were measured, as described in Hosseini, Brenig, et al. (2019). All individuals showed no caudal fin damage and exhibited normal morphology. The real sex in the control and temperature treatment groups was individually determined in adult fish by microscopic inspection. All husbandry facilities, fish management and water quality control, animal care and feeding, and data recording are described in detail by Hosseini, Brenig, et al. (2019).

### 2.3 Image acquisition

For imaging of fish, each adult individual was removed from their tank and killed in ice water. The fish was then placed in a glass petri plate and photographed. The images of all adult fish were captured individually using a digital camera (Nikon D7200). The Nikon D7200 is an Advanced Photo System type‐C (APS‐C) digital single‐lens reflex camera with a maximum image resolution of 6,000 × 4,000 (24 megapixels). This camera has an AF‐S DX Micro‐NIKKOR 40 mm 1:2.8G lens with high resolution and contrast from infinite to full size (1:1 magnification). For taking the pictures, the camera was fixed in a Kaiser RS3 Copy Stand (kaiser‐fototechnik) at a distance of 17 cm from the object location, at which fish were placed in a glass petri plate. In order to provide the optimal conditions for eliminating specular reflections and shadow problems, two different lighting techniques were applied. First, a light panel; Rex‐Slimline (Rex Leuchtplatten) was used for upward illumination, and second, a Cold LED Light Source; KL 300 LED (SCHOTT AG Lighting and Imaging) was used for lateral illumination. All photographs were taken vertically from the lateral view of fish in an ordinary fish laboratory room under common fluorescent light and under a well‐defined setting as described above. The same image recording procedure was used for all individuals in this study. All images were saved in RAW file and TIFF format with a resolution of 4,496 × 3,000 pixels. RAW files were then imported into the Photoshop software (Adobe Photoshop CS5, Photoshop Extended, version 12.0, 2010), for processing. The whole caudal fin region was cut out for SVM analysis. The caudal fin region was defined by the base of the fin at the caudal end of the body from the proximal to the distal end, which was clearly visible in the picture. We used one image of each of 448 animals to determine the sex. In an image preprocessing step, one out of 448 images was discarded from the dataset used for sex classification by machine learning due to low picture quality.

### 2.4 Color feature extraction

**Figure 1:**
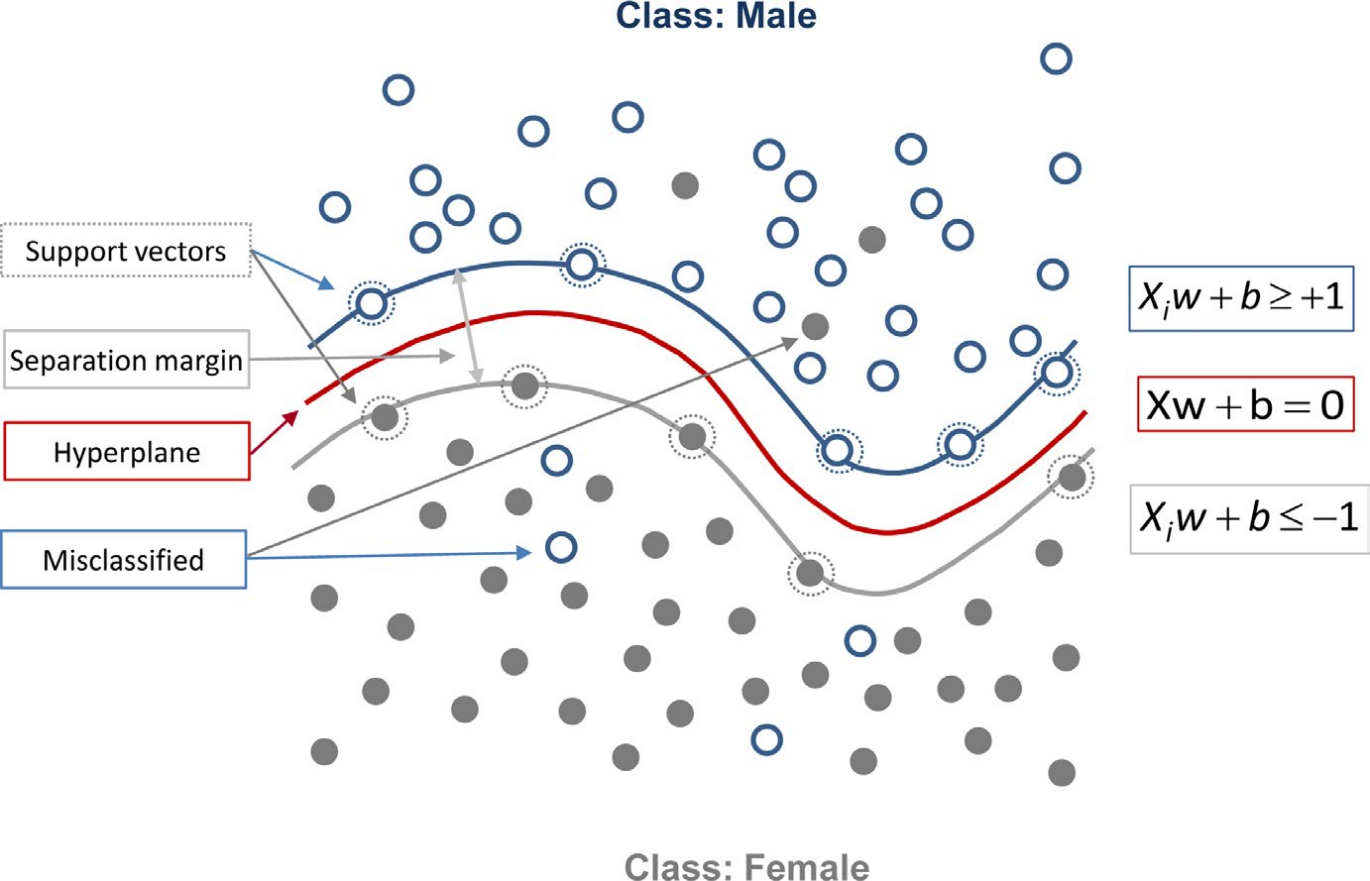
The schematic diagram represents the Support Vector Machine classification with Gaussian kernel function used in this study for sex discrimination. *X_i_* presents samples (input data), *w* presents weights, and *b* illustrates the bias factor

Sex classification of the whole fish body and caudal fin was conducted using DCNNs and SVM. For the color‐based classification of sex, color histograms (Swain & Ballard, 1991) were used to train a SVM (Keerthi & Lin, 2003). To do so, the images were first transformed from the RGB color space to the laboratory color space. Based on the converted laboratory image, histograms to describe the color distribution for each channel were calculated. For this purpose, all the colors in each image were counted and a frequency distribution generated. These histograms were used as features to train the SVM with a Gaussian kernel. A simple illustration of SVM with Gaussian kernel can be found in Figure 1. The main idea of training the SVM was to obtain a hyperplane that could separate the histograms in order to enable sex classification. The maps transform the histogram to a score value by calculating the distance from the histogram in a higher dimension to the hyperplane, giving a scalar value, which is given the L2 (Euclidian) distance (see materials and methods in Appendix S1).

### 2.5 Deep convolutional neural networks

**Figure 2:**
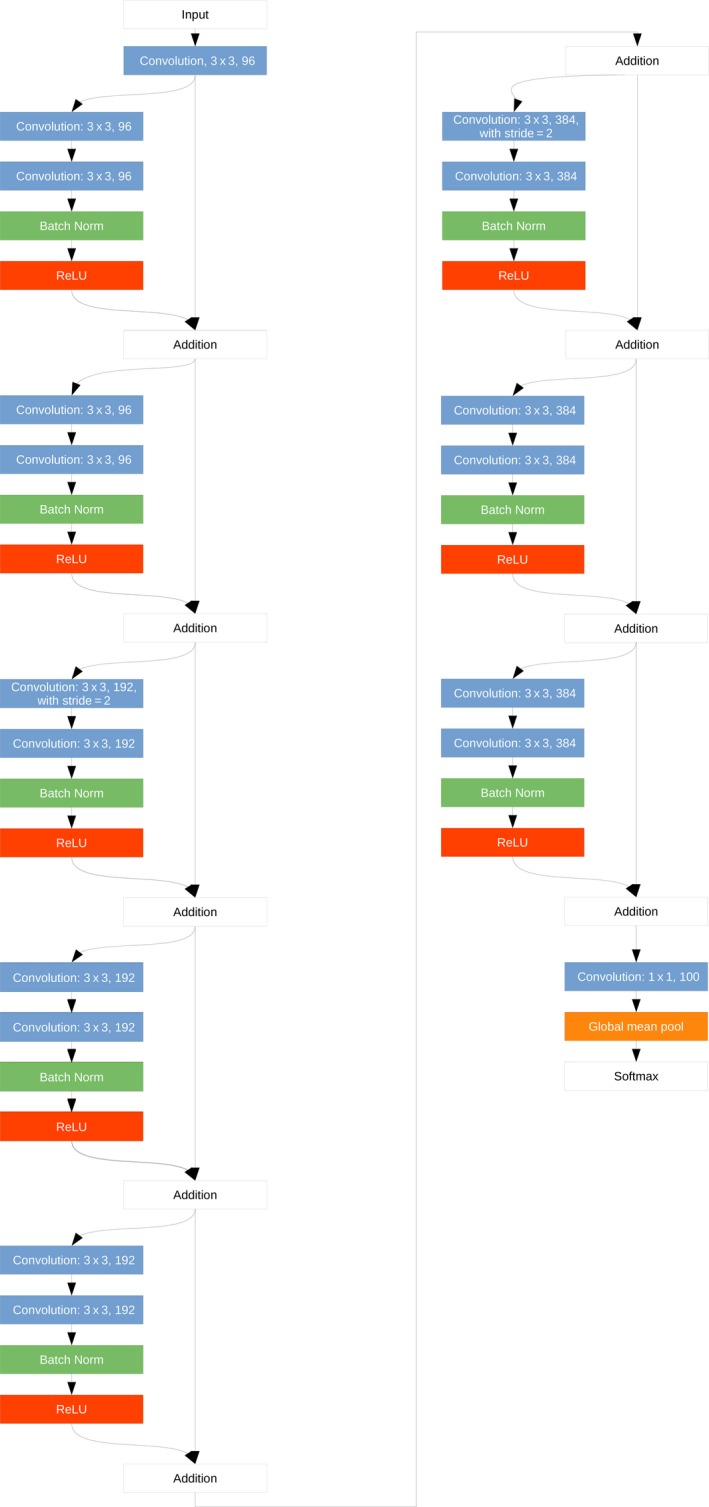
The graph represents the flow chart of the architecture of the convolutional neural networks (CNNs) applied for sex classification in this study

A convolutional neural network is an artificial neural network introduced by Krizhevsky, Sutskever, and Hinton (2012). Basically, the structure of a classical CNN consists of one or more convolutional layers, followed by a pooling layer. In principle, this unit can be repeated as often as required; with sufficient repetitions, this is referred to as DCNNs. The data processing through a network is done layer wise. The flow chart of the CNN architecture applied for sex classification in this study is illustrated in Figure 2. This CNN uses raw image pixels to model a simple differentiation score function. The architecture consists of the layers, which are arranged in three dimensions: width, height, and depth, where width and height are the dimensions of the image, and depth represents the color channels: red, green, and blue. The original image is transformed layer by layer from the original pixel values to the final class score. As mentioned before, the INPUT layer receives the raw pixel values of an image as a matrix. In this case, an image is a tensor with the size (255 × 255 × 3). The convolution layers calculate a point product from the comparatively small convolution matrix (also called filter kernel) with the currently underlying image section, whereby the convolution matrix moves stepwise over the input of the layer. For the first convolution layer, we used 96 convolution matrices (filters) of 3 × 3 pixels. In the next steps, the number of filters was increased to 384. After normalization, the convolution input of each neuron was transformed by a Rectified Linear Unit (ReLU) activation function (Nair & Hinton, 2010) into the output that models the relative significance. To prevent the vanishing of the gradient during training residual connections were used (He, Zhang, Ren, & Sun, 2015), where the outputs of one layer are re‐added to the original input matrix. This process was repeated in a consecutive processing using the increased number of filters. To minimize overfitting, the last feature layer was reduced by a global mean pooling operation. At the end, the probabilities for a binary outcome, namely the sex, were determined. For this purpose, the Softmax function was used, which represents the probability distribution over different possible events (see materials and methods in Appendix S1).

### 2.6 Statistical analysis

Statistical analysis of treatment effect on phenotypic sex of adult zebrafish was performed by applying a linear logistic model with a binary response variable, which was modeled as a Bernoulli random variable with *y_i_*. The dependent variable (*y_i_*) can take the value 1 with the probability of being male *π_i_* or 0 with the probability of being female 1−*π_i_* for observation *i*.

The logistic model uses a link function *g* (*π_i_*) linking the expected value to the linear predictors *η_i_*.

The data were then analyzed with the GLIMMIX procedure of SAS according to the following model:

$$Logit\left(\frac{\pi_{i}}{1-\pi_{i}}\right)=\eta_{i}=\mu +\alpha_{i}$$

where *π_i_* is the probability to be male, *μ* is the overall mean effect, *α_i_* is the fixed effect of temperature treatment (*i* = 1: temperature‐treated eggs 35°C, *i* = 2: control group 28°C). Least squares means were estimated on the logit scale and then back‐transformed to the probability scale using the inverse link function $\pi_{i}=\exp\left(\eta_{i}\right)/\left[1+\exp(\eta_{i})\right]$, applying the LSMEANS statement. Significant differences between least squares means were tested using a *t* test procedure by inclusion of the PDIFF option in the LSMEANS statement and adjusted by Tukey–Kramer correction. Standard errors of least square means were calculated as described by Littell, Milliken, Stroup, and Wolfinger (2006).

The impact of treatment and sex on body weight and length were analyzed using the GLM procedure of SAS with the following model:

$$y_{\mathrm{ijk}}=\mu +\alpha_{i}+\beta_{j}+\alpha \beta_{\mathrm{ij}}+\varepsilon_{\mathrm{ijk}}$$

where *y_ijk_* is the observation for body weight and length, *μ* is the general mean, *α_i_* is the effect of treatment (temperature treatment, control), *β_j_* is the fixed effect of sex, *αβ_ij_* is the fixed effect of interactions between treatment and sex, and $\varepsilon_{\mathrm{ijk}}$ is the random error.

To determine the degree of association between the sex classifications using DCNNs and SVM with the real sex in a further analysis, the mean square contingency coefficient (phi‐coefficient; *φ*) was estimated using the FREQ procedure of SAS through the construction of contingency tables.

The SVM estimates are individual scores for being male or female based on RGB color, which was used for sex classification. The variation of the differences between these estimated scores for males and females reflects the degree of color intensity. An individual with a higher score for a certain sex, which is dependent on the color intensity, was classified as male or female. We created a new variable in the first step based on the differences between the estimated scores of males and females for each individual. Then, we computed the *z*‐score of this variable by applying the *z*‐transformation, which was used as a dependent variable in the statistical model in order to analyze the association between body weight and length and the degree of caudal fin pigmentation for the different sexes, treatments, and their interactions as main factors. For this purpose, an analysis of covariance was applied using body weight and length as the covariate terms with up to three polynomial degrees, and considering the fixed main factors (real sex, treatment) and their interaction effects as well as the interactions between the main factors and the covariate (body weight or length) up to degree 3 of the polynomial. The final model was obtained by backward elimination of nonsignificant factors and factor combinations using *F*‐statistics. Since extreme observations or outliers can influence the parameter estimations, outliers of the dataset were detected using the influence diagnostics recommended by Belsley, Kuh, and Welsch (1980). The influence diagnostics methods are incorporated into the REG and MIXED procedure of SAS by using the INFLUENCE option in the MODEL statement (SAS/STAT^®^ 9.2 User's Guide, the MIXED Procedure, 2008). The Studentized residuals are excellent statistics for detecting unusual observations. We estimated the Studentized residuals using the INFLUENCE option in the MODEL statement of SAS' mixed procedure to estimate residuals for each recorded data point. 20 records out of the 448 data points with absolute extreme values larger than 3 (Hosmer, Lemeshow, & Sturdivant, 2013) were identified and discarded from subsequent statistical analysis. The final statistical model was applied with the mixed procedure of SAS as follows:

$$y_{\mathrm{ijk}}=\mu +\alpha_{i}+\beta_{j}+\alpha \beta_{\mathrm{ij}}+b_{1}\left(x_{\mathrm{ij}}\right)+b_{2}\beta_{j}\left(x_{\mathrm{ij}}\right)+\varepsilon_{\mathrm{ijk}}$$

where *y_ijk_* is the quantile of the standard normal distribution (*z*‐score) as described above, *μ* is the general mean, *α_i_* is the effect of treatment (temperature treatment, control), *β_j_* is the fixed effect of sex, *αβ_ij_* is the fixed effect of an interaction between treatment and sex, *b*_1_ is the linear regression coefficient of body weight or length (*x*), *b*_2_ is the linear regression coefficient of interaction between sex and body weight or length (*x*), and $\varepsilon_{\mathrm{ijk}}$ is the random error. SAS system version 9.3 (SAS Institute Inc. 2014) was used for all aforementioned statistical analyses.

## 3 RESULTS

### 3.1 Sex classification detected via machine learning methods

**Figure 3:**
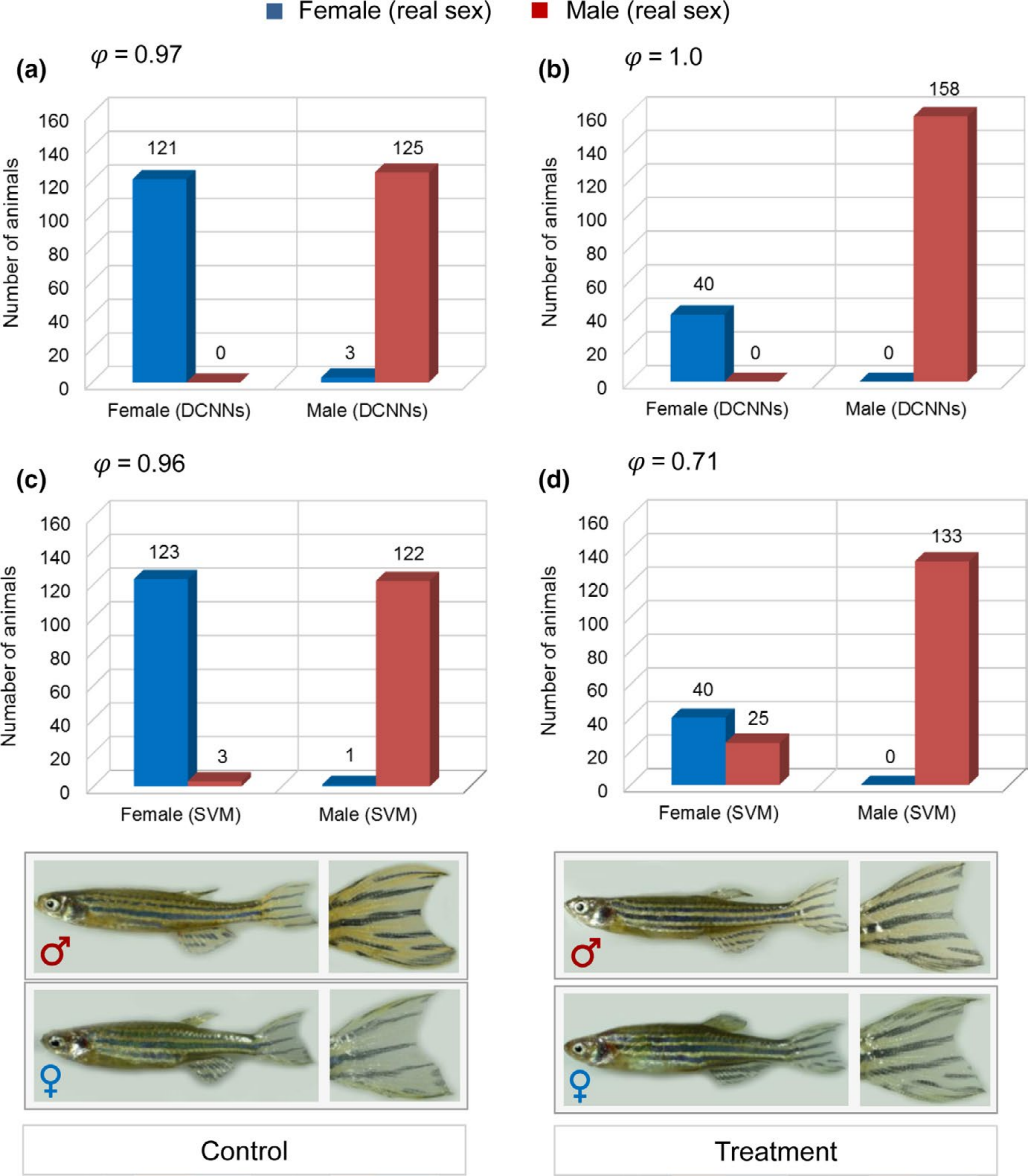
The result of sex classification using deep convolutional neural networks (DCNNs) and support vector machine (SVM) methods compared with real sex. The degree of agreement (*φ*) between sex classification using DCNNs analysis of adult fish body features and SVM analysis using color of caudal fin with real sex in control (a, b) and temperature treatment groups (c, d)

For evaluating the classification performance of the two machine learning methods, the result of these methods was compared with the real sex output in the different experimental groups. The descriptive statistical analysis of the real sex using microscopic inspection of gonadal tissues showed that high temperature resulted in an increase in the proportion of males compared to the control group (79.80% vs. 50.20%). These differences were highly significant by applying generalized linear model (*p* < .0001). The detailed results of this part of the experiment are given in Hosseini, Ha, et al. (2019). According to the DCNNs analysis, the percentage of male was 79.80% in the heat‐treated group versus 51.41% in the control group. The descriptive statistic for SVM showed 67.17% male in the treatment compared to 49.40% in the control group. The result of the generalized linear model analysis revealed that these differences were also highly significant for both machine learning approaches. Classification of sex using DCNNs and SVM methods and the degree of association with the real sex are presented in Figure 3. The DCNNs were trained to classify the sex based on color and pattern of fish pictures, while SVM classified the sex based on the caudal fin color only. Our analyses demonstrated a high agreement (*φ* = 0.97) between the sex ratio determined by DCNNs and the real sex ratio in the control group. The same applies to sex determination using SVM in the control group, which showed a slightly lower association with the real sex (*φ* = 0.96; Figure 3a,b). In temperature‐treated animals, DCNNs were able to determine the sex in complete agreement with the real sex (*φ* = 1.0), whereas sex classification by color features in the caudal fin using SVM showed a lower association with the real sex (*φ* = 0.71; Figure 3c,d). In this analysis, 25 animals were misclassified as females using SVM, all of which were temperature‐treated male animals. Inspection of the pictures of those animals showed a lower caudal fin pigmentation intensity compared to the regular males in the control group. To investigate this further, we performed a new analysis considering the color intensity of these animals resulting from SVM as a new level of the main factor sex and compared them with the color intensity score of correctly classified males in the temperature‐treated group, which resulted in a significant difference in color intensity between these two groups (−0.3526 vs. 0.6004; *p* < .0001). We suppose these animals were neomales who had altered their sex from genotypic females to phenotypic males in response to high water temperature and showed lower pigmentation intensity due to the temperature‐induced masculinization.

### 3.2 Association between color intensity and body weight and length

**Figure 4:**
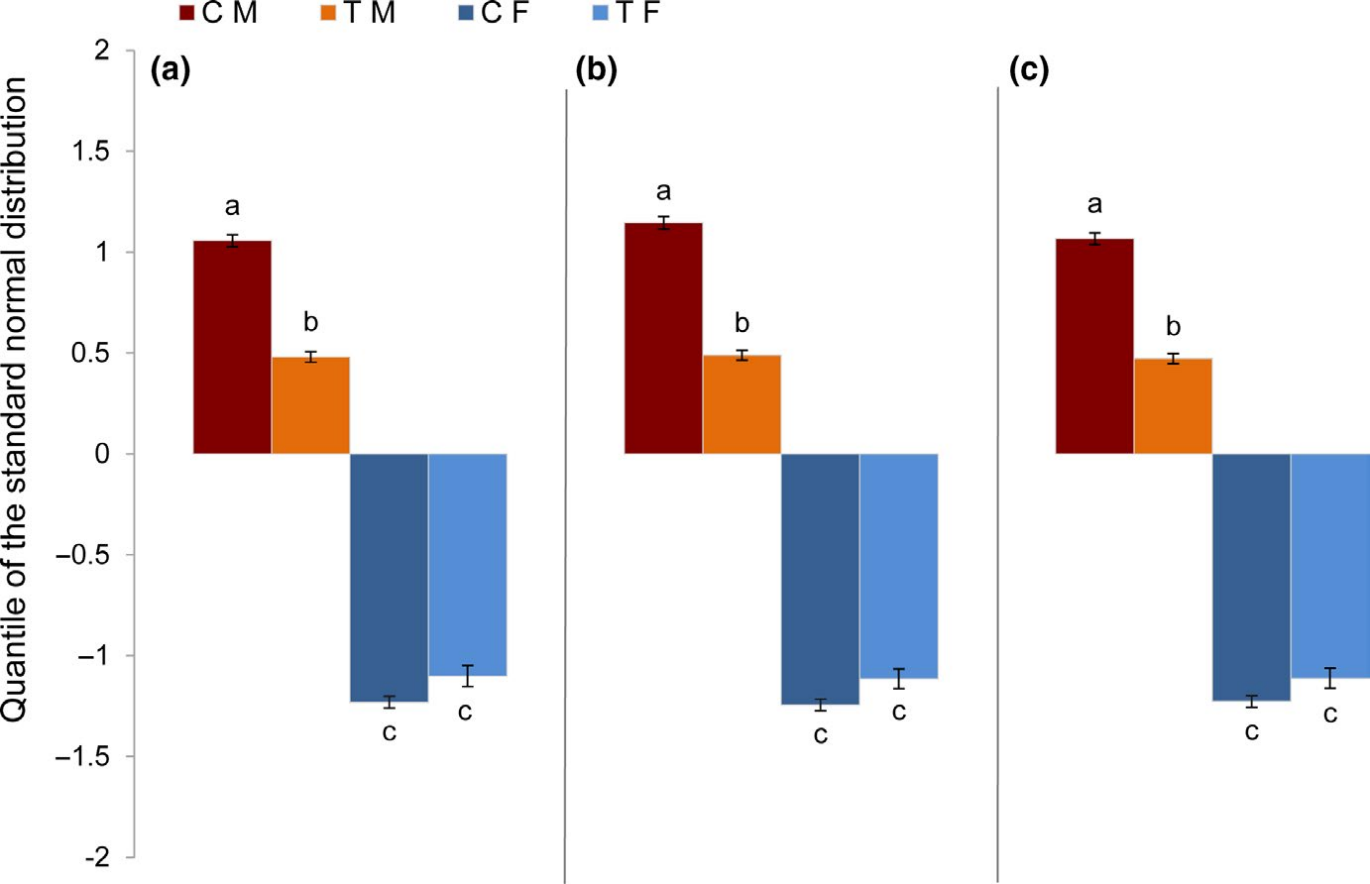
Association between degree of caudal fin coloration using SVM with body weight and length in different experimental groups: CF (control female), CM (control male), TF (treatment female), and TM (treatment male). (a) LS‐means for the levels of Treatment × Sex interaction without considering any covariates in statistical model and considering the covariate of body weight (b) and total length (c). Different alphabets (a–c) illustrate the significant differences between the least squares means of different factor levels (*p* < .0001). *Y* axis represents the color intensity of caudal fins derived from SVM

**Figure 5:**
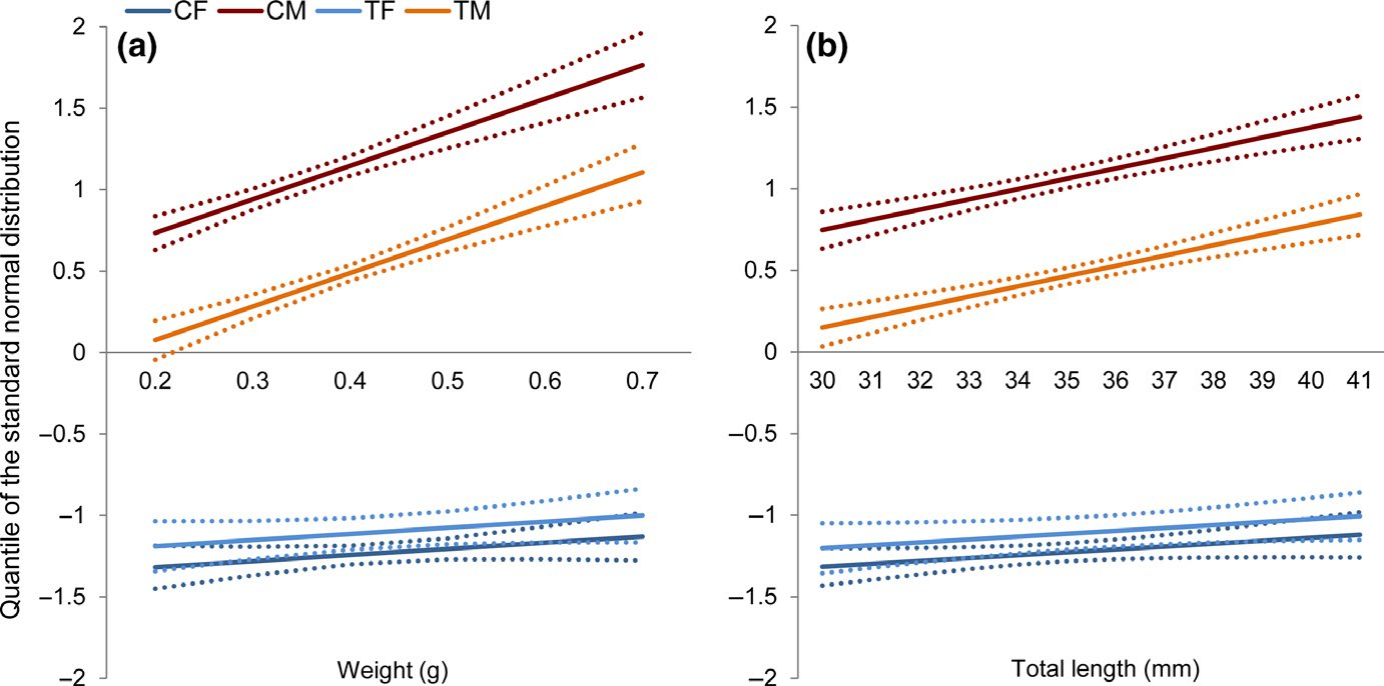
The effect of body weight (a) and total length (b) on pigmentation intensity in different experimental groups: CF (control female), CM (control male), TF (treatment female), and TM (treatment male). The solid lines show the LS‐means at certain level of body weight and total length. The dash bars present the confidence limits of LS‐means. *Y* axis represents the color intensity of caudal fins derived from SVM

In a further stage of this study, the degree of caudal fin coloration in association with body weight and length for the fixed effects of sex, treatment and their interactions were analyzed (Figures 4 and 5). As expected, the sex as a main factor in the statistical model leads to distinct differences in pigmentation intensity between males and females without considering any covariates (body weight or length) in the statistical model. Males showed a higher pigmentation intensity compared to females (male: 0.768 vs. female: −1.165). Considering the main factor treatment, the treated animals exhibited a lower pigmentation intensity compared to the control group (treatment: −0.0869 vs. control: −0.3102), in response to high water temperature. We found a significant sex–temperature interaction on pigmentation intensity. Comparing the pigmentation intensity of females without considering any covariates in the control and treatment groups revealed that there is no significant difference in coloration between these two groups. In contrast to this, high temperature treatment resulted in a distinct reduction in color intensity of males, which partly contributed to a significant treatment effect (Figure 4a). This result indicates that temperature treatment during early embryogenesis might have an impact on the expression of pigmentation genes responsible for development of the caudal fin coloration and phenotypic variation in males. The same is true considering the adjusted means derived from the main factors and interactions using body weight or length as covariates (Figure 4b,c). The result of the analysis of covariance also showed a significant positive association between body weight and length of adult fish with the degree of caudal fin color intensity in males both in the control and heat‐induced groups, whereas no significant association was detected in females (Figure 5a,b). To the best of our knowledge, this finding has not been reported in zebrafish so far.

### 3.3 Body weight and length

Table 1 summarizes the least squares means and the significance of explanatory variables on body weight and total length in adult zebrafish. The main factor treatment and sex as well as their interactions (treatment × sex) showed a significant influence on body weight. However, only a significant influence of the main factor sex on length was observed. Zebrafish raised at a high temperature (HT) were heavier (0.4161 vs. 0.3952) and longer (35.4976 vs. 34.8982) than those raised at a low temperature (LT) condition. In the case of sex, a significant difference of body weight was observed between males and females; a considerably higher body weight was found in the female group versus males, while the effect on total length was not significant. The differences in body weight between LT‐ and HT females for the effect of interaction between treatment × sex were not significant. However, temperature treatment resulted in a significant reduction of body weight in males.

**Table 1:** Least squares means, standard error (±*SE*), and ANOVA significance level for body weight (g) and total length (mm) in adult zebrafish for the effect of temperature, sex, and treatment × sex interaction

Effect		Traits	
		Body weight	Total length
Treatment			
Low temperature^1^ (LT)		0.3952 ± 0.0049^a^	34.8982 ± 0.1323^a^
High temperature^2^ (HT)		0.4161 ± 0.0067^b^	35.4976 ± 0.1816^b^
Sex			
Male		0.3773 ± 0.0047^a^	35.1049 ± 0.1253^a^
Female		0.4339 ± 0.0069^b^	35.2909 ± 0.1865^a^
Treatment × Sex			
LT × Male		0.3582 ± 0.0070^a^	34.9648 ± 0.1882^a^
HT × Male		0.3965 ± 0.0062^b^	35.2450 ± 0.1653^a^
LT × Female		0.4321 ± 0.0069^b^	34.8316 ± 0.1859^a^
HT × Female		0.4357 ± 0.0121^b^	35.7502 ± 0.3233^a^

ANOVA significance level [*p* (*F*)]	Treatment	Sex	Treatment × Sex
Body weight	0.0131	<0.0001	0.0392
Total length	0.0079	0.4082	0.1560

Note

Different alphabets (a–c) illustrate the significant differences between the least squares means of different factor levels (*p* < .05).

^1^ Low temperature refers to the control group at 28°C.

^2^ High temperature refers to the temperature treatment group at 35°C.

## 4 DISCUSSION

Machine learning approaches recently became the leading technique for object and action recognition in humans (Jain, Tompson, Andriluka, Taylor, & Bregler, 2013; Toshev & Szegedy, 2013). The technique has great potential in animal sciences for studying of different aspects of animal behavior such as movement, food intake, social structure and competition, reproduction behavior, communication and welfare, and nesting using complex datasets (Borchers et al., 2017; Stern, He, & Yang, 2015; Valletta, Torney, Kings, Thornton, & Madden, 2017; Wang, 2019; XU & Cheng, 2017). Automated imaging technologies provide a large number of images that require an efficient strategy of analysis such as machine learning. Thus, image analyses can be used to identify and classify objects in various biological research aspects (Grys et al., 2016; Liakos et al., 2018). The study of Jeanray et al. (2015) is an example of image analysis for classifying phenotypical deformity in zebrafish larvae using machine learning methods. Their study on phenotypic classification of images resulted in a high agreement with manual classification by biological experts. Automation of analysis and classification of sex using machine learning methods directly from images is a great possibility to increase the efficiency in the prediction process (Singh & Goel, 2016). The current guidelines for phenotypic sex classification in zebrafish using conventional methods are subjective and rely on different phenotypic observations by humans, which are highly labor‐intensive and potentially prone to error in the prediction process (McMillan et al., 2015). For example, zebrafish quickly become pale after removing from the water (Hutter et al., 2010), which may refer to the presence or absence of iridophore pigment cell types. Iridophores give the body a shiny appearance by reflecting light in the water (Frohnhöfer, Krauss, Maischein, & Nüsslein‐Volhard, 2013; Patterson & Parichy, 2013). Iridophores are present in the body coloration, but are not involved in the fin color formation (Singh & Nüsslein‐Volhard, 2015). It is often difficult to distinguish between sexes using different body colors outside of water. In this study, we presented two fully automated machine learning methods (DCNNs and SVM) as efficient, robust, and flexible approaches for sex classification in zebrafish using a set of images for the first time in a color space designed for human perception. Machine learning classification relies on the quality, size, and objectivity of learning datasets, which is more reliable than manual approaches for sex determination in zebrafish. Our method is not specific to zebrafish; it is a general approach that could be applicable to other organisms for future research. Based on phenotypic characteristics in this study, a high accuracy of sex differentiation was obtained using these two methods in nonheat‐treated groups.

Furthermore, in this study, we reported a less intense color in the caudal fin of a subset of adult zebrafish using SVM who were exposed to elevated water temperature during embryogenesis. Based on this result, classification of sex using SVM technique is applicable for sexing in nonexperimentally manipulated individuals, but it may be less effective when the phenotypes (color) are experimentally altered. Therefore, some treated males in this study were misclassified using SVM due to reduced pigmentation intensity. However, DCNNs was able to classify the sex with high performance independent of the alteration of phenotypes.

The effect of high temperature on the loss of pigmentation has already been observed in adult zebrafish in another study, where the animals were thermally influenced during the larval stage (Ribas et al., 2017). Since in our study, the pigmentation deficiency was mainly observed in heat‐treated males, suggesting that these animals might have been sex‐reversed or masculinized females. Therefore, it was hypothesized that decreased color intensity in masculinized animals would potentially influence sexual attractiveness and, thus, mating success. The molecular genetic study of heat‐induced masculinization in European sea bass showed that masculinization process is caused by epigenetic modifications (Navarro‐Martin et al., 2011). In a recent study, transcriptome analysis was used to distinguish neomales from the normal male zebrafish (Ribas et al., 2017). Hence, genetic and epigenetic investigations can be used to discriminate the masculinized from normal animals and to elucidate the physiological mechanisms of sexual‐reversed males in interaction with environment. However, the underlying mechanism of color intensity in interaction with the environmental temperature is still not clear and deserves further research.

Sexual selection represents a mode of selection on certain traits to increase an individual's reproductive success. Regarding sexual selection theory, males of many species develop a variety of secondary sexual characters and traits, such as body ornaments and pronounced coloration, to signal their attractiveness as mating partners (Uusi‐Heikkilä, Böckenhoff, Wolter, & Arlinghaus, 2012). In zebrafish, females usually have a rounder shape than males, and males display a more intense yellow coloration than females particularly during sexual activity (Gerlai, Lahav, Guo, & Rosenthal, 2000; Hutter et al., 2010; Singh & Nüsslein‐Volhard, 2015), which appears to be important for sexual attraction. Since reproduction is energetically costly it is generally expected that there is a positive relationship between partner quality and reproductive investment in terms of differential allocation theory (Uusi‐Heikkilä et al., 2012). In a study on a laboratory strain of zebrafish, neither female preferences nor spawning successes were associated with the male body size (Spence & Smith, 2006). Furthermore, the effect of male body size for female preferences could not be detected because the hypothesis of the study was focused on differences in male dominance, not on male body size with a large size variation (Spence & Smith, 2006). In contrast, other studies demonstrated a clear female preferences for larger males (Pyron, 2003; Skinner & Watt, 2007; Uusi‐Heikkilä et al., 2012). In these studies, females allocate their reproductive resources to larger males, characterized by a higher spawning probability and clutch size. In our study using machine learning techniques, we found a pronounced secondary sexual characteristic (color) in zebrafish males compared to females. In addition, a positive association between color intensity and body weight and length was observed in male zebrafish. Our results in this study suggest that male coloration and its positive association with body weight and length may play a role in sexual attraction in the zebrafish strain studied here and may increase reproductive success. However, whether caudal fin coloration and its interaction with body size serves as a sexually selected trait for mating success in this species requires further research. Sexual preferences using coloration and size have been observed in other fish species. For example, mating preference in *C. cyanea* is related to male body size and orange caudal fin color intensity (Gronell, 1989; Wacker, Östlund‐Nilsson, Forsgren, Newport, & Amundsen, 2016) and in *G. aculeatus* to redness of the throat in males (Kraak et al., 1999).

Another aspect of this study was to investigate the impact of elevated water temperatures on growth performance. The high temperatures during embryonic development positively influenced both traits of growth, namely body weight and length. Schnurr, Yin, and Scott (2014) also observed a higher growth rate in zebrafish treated with high temperature during embryonic development compared to the control group. Our previous study confirms this too; a positive effect of increased temperature during embryogenesis on growth rate of another zebrafish strain was observed, which was more pronounced in females than in males (Hosseini, Brenig, et al., 2019). Embryonic temperature in zebrafish affects thermal acclimation of muscle tissue, which has an influence on energy metabolism and swimming performance in adult fish, due to differences in the expression of genes involved in energy metabolism, cell stress, muscle contraction, and apoptosis (Scott & Johnston, 2012). Effects of temperature during embryonic development on growth rate were also reported in other fish species such as gilthead sea bream. Exposure to different temperature (low temperature: 18°C, high temperature: 22°C) during embryogenesis in sea bream and its effect on muscle growth rate and body weight revealed that early temperature treatment has an influence on the expression profiles of a part of muscle developmental genes (*Hsp90a*, *UNC45*, *MyoD*, and *IGF1*) and their expression is influenced by different temperature treatment (Serrana et al., 2012). In our study, we found a positive effect of increased temperature on growth performance in adult fish in response to high ambient temperature, which may partly be due to the effect of temperature on muscle developmental gene expression in zebrafish.

Generally, developmental plasticity can be influenced by many different environmental factors, which induce the expression of different phenotypes. A classic example of plastic responses to environment is the effect of temperature on different phenotypic traits such as sex determination, body size, pigmentation, and survival in many animal species (Lafuente & Beldade, 2019). Some plastic characteristics are simultaneously influenced by the same environmental factor and change in combined form in response to the environment. Some of such plastic traits might be adaptive, while some others might be maladaptive. These plastic responses to the environmental cues lead to phenotypic variation and diversification in a population, which affects response to selection (Lafuente & Beldade, 2019). However, phenotypic expression may be unfavorable as a result of plasticity under certain environmental conditions and only a subpopulation with certain phenotypic expression may adapt. Thus, the survival of an adapted subpopulation only could lead to an impoverishment of genetic variation, or in general might have unfavorable effects and the species could be threatened with extinction (Brown et al., 2015; Bürger & Lynch, 1995). As shown in this study, the correlated plastic responses of phenotypic traits, including sex determination and color to high temperatures, might contribute to population dynamics and a risk of extinction in changing environments (Allendorf & Luikart, 2007; Bürger & Lynch, 1995), particularly under extreme and fluctuating environmental conditions, which is relevant from an ecological point of view.

## 5 CONCLUSIONS

Phenotypic classification of sex using machine learning methods in this study resulted in a high degree of association with the real sex, which possesses the advantage of automatization and robustness with a high degree of accuracy compared to conventional methods. In addition, we found that high ambient temperature leads to a lower color intensity in some treated males, which was quantified by SVM in the caudal fin, suggesting that these animals were likely masculinized animals or neomales. Our findings further indicated that the color intensity is associated with body weight and length in males but not in females, which appears to play a significant role in sexual attractiveness. Furthermore, none of our techniques and developed software in this study is specific to zebrafish; the same approach can be readily applied to other species with phenotypic sexual dimorphism.

## CONFLICT OF INTEREST

None declared.

## AUTHOR CONTRIBUTIONS

SHo contributed to the conception and design of the study, carried out the experiments, interpreted the results, and wrote the manuscript. ARS substantially designed the study and conception. ARS and SHo performed computational data analysis and interpreted the data. SHe performed image analysis using DCNNs and SVM methods. ARS, HS, SHe, BB, and JT edited and corrected the manuscript. All authors read and commented on the manuscript and approved the final version.

## DEVELOPED SOFTWARE

The software for sex classification in this study is freely available in https://hdl.handle.net/21.11101/0000-0007-D837-8.

## Data Availability

The datasets used and analyzed in this study are included in the article and are available in Supporting Information (Data S1‐S2). Uses of dataset are in permission of corresponding author.
