# Supplementary material for: Efficient phenotypic sex classification of zebrafish using machine learning methods
Source: Ecol Evol. 2019 Nov 11;9(23):13332–43. doi: 10.1002/ece3.5788 (PMC6912926; doi:10.1002/ece3.5788)
Supplement: Supplementary file 2 [file ECE3-9-13332-s002.docx]

**Appendix S1**

**MATERIALS AND METHODS**

**1.1 | Color feature extraction**

For the color-based classification of sex, the images were first transformed from the RGB color space to the lab color space. Given the chromaticity coordinates of an RGB system $\left( x_{r},y_{r} \right),$ $\left( x_{g},y_{g} \right)$ and $\left( x_{b},y_{b} \right)$ together with its reference white $\left( X_{W},Y_{W},Z_{W} \right)$ such that

$$\left[ \begin{matrix} X \\ Y \\ Z \end{matrix} \right]=M\left[ \begin{matrix} R \\ G \\ B \end{matrix} \right]$$

where

$M=\left[ \begin{matrix} S_{r}X_{r} & S_{g}X_{g} & S_{b}X_{b} \\ S_{r}Y_{r} & S_{g}Y_{g} & S_{b}Y_{b} \\ S_{r}Z_{r} & S_{g}Z_{g} & S_{b}Z_{b} \end{matrix} \right]$,

$$with X_{r}=x_{r}/y_{r},Y_{r}=1,Z_{r}=\left( 1-x_{r}-y_{r} \right)/y_{r},X_{g}=x_{g}/y_{g},Y_{g}=1,Z_{g}=\left( 1-x_{g}-y_{g} \right)/y_{g},$$

$X_{b}=x_{b}/y_{b},Y_{b}=1,Z_{b}=\left( 1-x_{b}-y_{b} \right)/y_{b}$ and

$\left[ \begin{matrix} S_{r} \\ S_{g} \\ S_{b} \end{matrix} \right]=\left[ \begin{matrix} X_{r} & X_{g} & X_{b} \\ Y_{r} & Y_{g} & Y_{b} \\ Z_{r} & Z_{g} & Z_{b} \end{matrix} \right]^{-1}\left[ \begin{matrix} X_{W} \\ Y_{W} \\ Z_{W} \end{matrix} \right]$.

This representation then can be used to calculate the lab color space by:

$$L=116\left( f_{y}-16 \right)$$

$$a=500\left( f_{x}-f_{y} \right)$$

$$b=200\left( f_{y}-f_{z} \right),$$

where

$f_{i}=\left\{ \begin{aligned} \sqrt[3]{i_{r}}& \text{if }i_{r}>\epsilon\\ \frac{\kappa i_{r}+16}{116}& \text{else} \end{aligned} \right.$ Where $i_{r}=\frac{X}{X_{r}}$ with $i \in\{x,y,z\}$ and

$\epsilon=0.008856$, $\kappa=903.3$ are defined by the CIE standards (McLaren, 1976). For every set of values of in $L,a,b$ a histogram is calculated, such that: $n=\sum_{i=1}^{k} m_{i}$ where $n$ is the number of observations and $k$ the total number of bins, for our study $k=255$. These histograms are used as features for a Support Vector Machine with a Gaussian kernel, we call the continuation of all three histograms $X_{i}$ with label $y_{i}$. In this way the training data is given as:

$\{\mathbf{X}_{i},y_{i}\}: i=\{1,\ldots,l\},\mathbf{X}_{i}\in\mathbb{R}^{n},y_{i}\in\{-1,1\}$.

This data is supposed to be separated by a hyperplane: $\mathbf{Xw} + b = 0$, where

- $w$ normal to the hyperplane
- $\frac{\left| b \right|}{\left| \left| \mathbf{w} \right| \right|}$ is the distance to origin
- $||\mathbf{w}||$ Euclidean norm of $\mathbf{w}$

Such that:

$$\mathbf{X}_{i}\mathbf{w}+b\geq+1, y_{i}=+1$$

$$\mathbf{X}_{i}\mathbf{w}+b\leq-1,y_{i}=-1$$

$y_{i}(\mathbf{X}_{i}\mathbf{w} + b) - 1 \geq0, \forall i$.

Since the distribution in the feature space of our data is not divisible by a linear hyperplane, we are assuming that:

$\boldsymbol{\Phi}:\mathbb{R}^{d}\to H$,

Such that there is a kernel $K$:

$K\left( x_{i},x_{j} \right)=\boldsymbol{\Phi}\left( \mathbf{X}_{i} \right)\boldsymbol{\Phi}\left( \mathbf{X}_{j} \right)$.

By choosing a Gaussian kernel we get:

$K\left( \mathbf{X}_{i},\mathbf{X}_{j} \right)=e^{\frac{-|\mathbf{X}_{i}-\mathbf{X}_{j}|^{2}}{2\sigma^{2}}}$.

**1.2 | Deep Convolutional Neural Networks**

The data processing through a network in convolutional neural networks is done layer wise. Considering $\mathbf{X}_{\alpha}\in\mathbb{R}^{h_{X}^{\left( 0 \right)}\times g_{X}^{\left( 0 \right)}}$ is an image with $h_{X}^{\left( 0 \right)}$ rows, $g_{X}^{\left( 0 \right)}$ columns, and $d_{X}$ channels (in our case of L, a, b channels $d_{X}=3$). Each channel of $\mathbf{X}_{\alpha}$ is denoted by $X_{\alpha}^{\left( k \right)}$, such that $\mathbf{X}_{\alpha}=\{X_{\alpha}^{\left( 1 \right)},X_{\alpha}^{\left( 2 \right)},\ldots,X_{\alpha}^{\left( d_{X} \right)}\}$. For each kernel $K^{l,d}\in\mathbb{R}^{h_{K}^{l}\times g_{K}^{l}}$ with size $h_{K}^{l}\times g_{K}^{l} and d\in\{1,\ldots,d_{K}\}$, the output $Y^{l}\in\mathbb{R}^{\frac{h_{X}^{l}-h_{K}^{l}-1+P^{l}}{S^{l}}\times\frac{g_{X}^{l}-g_{K}^{l}-1+P^{l}}{S^{l}}}$ is computed elementwise by:

$Y_{x,y}^{l,d}=\left( \mathbf{X}^{l}*K^{l,d} \right)_{x,y}=\left( b^{l,d}+\sum_{k=1}^{d_{X}^{l}} \sum_{i=1}^{h_{K}^{l}} \sum_{j=1}^{g_{K}^{l}} K_{i,j}^{l,d}\cdot X_{x+i-1,y+j-1}^{l,k} \right)$.
